# Risk and prognostic factors of replantation failure in patients with severe traumatic major limb mutilation

**DOI:** 10.1007/s00068-021-01876-w

**Published:** 2022-01-20

**Authors:** Chang Gao, Ling Yang, Jihui Ju, Ye Gao, Keran Zhang, Mingming Wu, Lijuan Yang, Xiaoting Lu, Ruixing Hou, Qiang Guo

**Affiliations:** 1 Department of Emergency and Critical Care Medicine, Dushu Lake Hospital Affiliated to Soochow University (Suzhou Dushu Lake Hospital), Suzhou, Jiangsu China; 2 Medical Center of Soochow University, Suzhou, Jiangsu China; 3 Medical College of Soochow University, Suzhou, Jiangsu China; 4 Department of Orthopaedic, Ruihua Affiliated Hospital of Soochow University (Suzhou Ruixing Medical Group), Suzhou, Jiangsu China; 5 Department of Critical Care Medicine, Taicang Affiliated Hospital of Soochow University, Suzhou, Jiangsu China; 6 Department of Critical Care Medicine, Ruihua Affiliated Hospital of Soochow University (Suzhou Ruixing Medical Group), Suzhou, Jiangsu China; 7 Department of Critical Care Rehabilitation Medicine, Suzhou Ruisheng Rehabilitation Hospital (Suzhou Ruixing Medical Group), Suzhou, Jiangsu China; 8 The First Affiliated Hospital of Soochow University, Suzhou, Jiangsu China

**Keywords:** Replantation, Major limb, Predictors, Risk factor

## Abstract

**Purpose:**

Traumatic mutilation of major limbs can result in limb loss, motor disability, or death. Patients who had replantation failure needed to undergo additional surgeries (even amputation) and had a longer length of hospital stay. Here, we determined the risk and prognostic factors of replantation failure in patients with traumatic major limb mutilation.

**Methods:**

This retrospective study included adult inpatients with severed traumatic major limb mutilation who underwent replantation from Suzhou Ruixing Medical Group from October 18, 2016 to July 31, 2020. Demographic, and clinical characteristics including traumatic conditions, laboratory findings, mangled extremity severity scores (MESS), treatments, and outcomes of the patients were collected. Data were used to analyze predictors and risk factors for replantation failure.

**Results:**

Among the 66 patients, 48 (72.7%) were males, the median age was 47.0 years old. Replantation failure occurred in 48 patients (72.7%). The area under the curve of the joint prediction of lactic acid on admission, 72-h cumulative fluid balance, and albumin level immediately postoperatively was 0.838 (95% confidence interval [CI], 0.722–0.954; *P* < 0.001) with a sensitivity of 89.7% and a specificity of 69.2%. Lower limb trauma (odds ratio [OR] 8.65, 95% CI 1.64–45.56, *P* = 0.011), mangled extremity severity scores (OR 2.24, 95% CI 1.25–4.01, *P* = 0.007), and first 72-h cumulative fluid balance > 4885.6 mL (OR 10.25, 95% CI 1.37–76.93, *P* = 0.024) were independent risk factors for replantation failure.

**Conclusions:**

Lower limb trauma, mangled extremity severity scores, and cumulative water balance were associated with replantation failure, implying that fluid management is necessary for major limb salvage. More studies are needed to explore the predictive power of indicators related to tissue oxygenation and wound healing for replantation failure.

**Supplementary Information:**

The online version contains supplementary material available at 10.1007/s00068-021-01876-w.

## Background

Traumatic mutilation of the major limbs is a life- and limb-threatening injury that may result in death, limb loss, or persistent functional motor disability [1–3]. A retrospective study in France showed that among 1715 patients with traumatic upper limb amputation between 2004 and 2013, the majority (84.6%) were male, and the common causes of injury were saw and crush [4]. Explosions are one of the leading causes of traumatic amputation of large limbs in war Settings, along with traffic accidents [5, 6]. Management involves bone fixation, interventional or surgical revascularization, and complex wound care that treats infections and segmental loss of bones, muscles, and nerves [1]. For patients with persistent infection, open wounds, and replantation failure, additional treatment via debridement, flap covering, and/or amputation is required. These problems can impose severe physical, psychological, financial, and social distress on the patients [7–9].

Previous studies have shown that age, high injury severity score, blunt trauma, injury location, duration of ischemia, and reperfusion injury are associated with replantation failure and delayed amputation [7, 10, 11]. With the development of treatment for vascular, bone, nerve, and soft tissue injuries, injury severity scores such as mangled extremity severity scores (MESS) have varied in predicting the prognosis of amputation and replantation [7, 12–15].

Microcirculation plays an important role in maintaining the homeostasis of end organs and regulating tissue perfusion [16]. It is vital to optimize intravascular volume to promote adequate oxygen delivery to shock tissues [17]. Most patients with major limb amputation are accompanied by hemorrhagic shock of varying degrees. Although fluid resuscitation can maintain circulation stability, fluid responsiveness was inconsistent between systemic and microvascular hemodynamics [18]. Management of severely injured limbs remains a major challenge [19]. Here, we identify the risk and prognostic factors of replantation failure in patients who had traumatic mutilation of major limbs.

## Methods

This was a multicenter retrospective cohort study. This study included all adult patients (age ≥ 18 years) who had traumatic mutilation of major limbs (defined as an amputation between the trunk and the wrist or ankle) and underwent replantation [20, 21] between October 18, 2016 and July 31, 2020 from three hospitals in the Suzhou Ruixing Medical Group (Ruihua Affiliated Hospital of Soochow University, Ruixing Hospital, and Suzhou Ruihua Yingchun Hospital). All enrolled patients were admitted to the intensive care unit (ICU) because of their critical conditions. The medical group includes Level III specialized hospitals, rehabilitation hospitals, and an institute of applied technology in hand surgery. The focus medical programs of the group are orthopedic trauma, amputated limbs (fingers and toes) replantation, and rehabilitation, with an average annual operation volume of more than 10,000. All of the mutilation limbs were accompanied by discontinuous vessels, nerves, muscles, and bone structures to varying degrees. Patients with severe limb damage that could not be replanted or had first-stage amputations were excluded. Patients who underwent replantation were identified by reviewing and analyzing admission logs and histories from all available electronic medical records and patient care resources.

Medical records were reviewed by trained physicians. Demographic and clinical characteristics of the patients were collected. Clinical characteristics included traumatic conditions, laboratory findings, MESS, 72-h cumulative fluid balance after admission, treatments, and outcomes. Laboratory findings included lactic acid level on admission; white blood cell count, neutrophil count, lymphocyte count, platelet count, red blood cell count, albumin level, blood urea nitrogen level, and creatinine level immediately after surgery. During hospitalization, clinicians performed blood routine, coagulation routine, liver function, kidney function, and other blood examinations according to the conditions. Patients were followed-up from admission to hospital discharge. The primary outcome was the replantation failure rate during hospitalization.

Indications for replantation of severed limb: (1) Relatively complete distal limb and mild skin contusion, (2) The tissue structure of the proximal limb is relatively complete, and the bone and joint injury does not seriously affect the appearance and function of the limb, (3) No avulsive nerve injury or only minor local contusion, (4) Patients could tolerate microsurgery with stable physical signs and without serious complications. Replantation failure was identified in patients with signs of any partial/total necrosis or capillary refill loss [22]. Delayed amputations were defined as amputations performed within the same hospitalization period after replantation [23]. All patients with replantation failure required additional surgery at least once. The secondary outcomes were the length of ICU stay and hospital stay.

Frequency data were expressed as proportions. Continuous data are presented as median (interquartile range [IQR]) if they showed skewed distribution. Shapiro–Wilk test was used to determine normal/skewed distribution of the data. Differences in categorical variables were assessed using the *χ*^2^ test, while comparisons of continuous variables were made using the Mann–Whitney *U* test, as appropriate. Receiver operating characteristic (ROC) curves were used to explore the predictors and their cutoff values for replantation failure. The area under the curve (AUC) of ROC was used to evaluate predictive power. Multivariate logistic regression models were constructed to obtain the prediction probability while the AUCs of the ROC were used to evaluate the effectiveness of the combined predictions.

Multivariate logistic regression models were used to determine the independent risk factors for necrosis after replantation. The logistic regression results are presented as (odds ratio [OR], 95% confidence interval [CI]). Variables with *P* < 0.2 in univariate logistic regression were included in the multivariate analysis. The probabilities of entering and removing variables in a stepwise manner in the multivariate model were 0.05 and 0.10, respectively.

Data were analyzed using SPSS (version 25.0; IBM, Chicago, IL, USA). Statistical charts were generated using StataMP 16 (StataCorp, College Station, Texas, USA) and GraphPad Prism 7 (GraphPad Software, San Diego, CA, USA). A two-tailed *P* value of < 0.05 was considered statistically significant.

This study was approved by the Institutional Review Boards of the Suzhou Ruixing Medical Group (2021023).

## Results

### Clinical characteristics

**Fig. 1:**
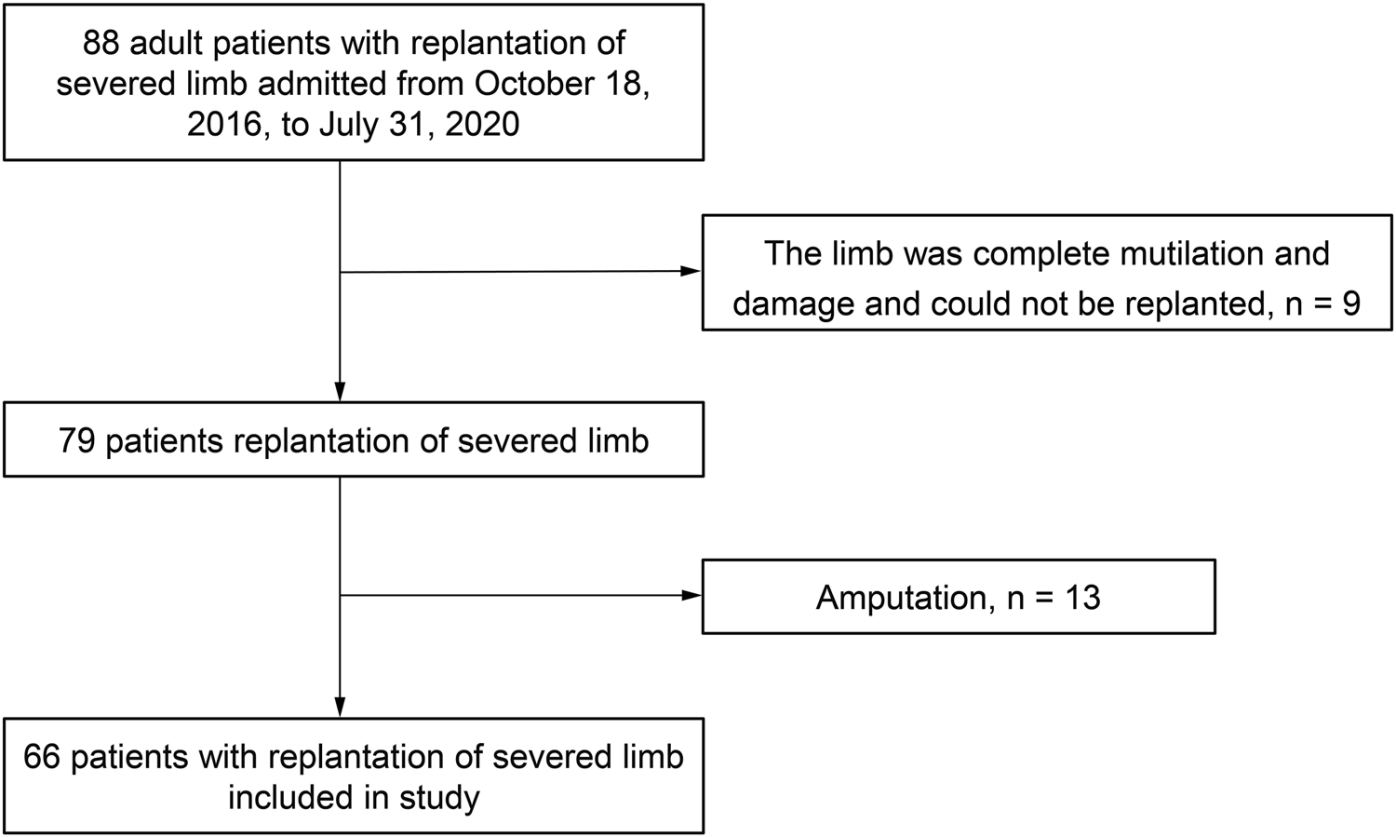
Study flowchart

During the 4-year study period, 88 patients were admitted to the hospital after experiencing traumatic major limb mutilation. A total of 22 patients who had either severe limb damage that could not be replanted or first-stage amputations were excluded. The remaining 66 patients who underwent replantation were included in this study (Fig. 1).

The median age of the cohort was 47.0 (IQR 36.0–54.5) years and the majority were males (*n* = 48, 72.7%). The distribution of patients in terms of injury was as follows: 64 (97.0%) had blunt trauma, 32 (48.5%) had lower limb trauma, and 29 (43.9%) had total mutilation. Most patients experienced blunt trauma with moderate-to-severe contamination. The median MESS was 10.0 points (IQR 9.0–11.3) (Tables 1 and 2). Replantation failed in 48 (72.7%) patients, of whom 41 (62.1%) had partial necrosis and 7 (delayed amputation rate, 10.6%) had whole limb necrosis. In the cases of replantation failure, 11 cases were complicated by bacterial infection at the surgical site, 7 had thrombosis, and 1 had vascular crisis. Of the 7 patients with delayed amputation, 3 were upper limbs and 4 were lower limbs. The upper limb salvage rate was 91.2% (31 in 34 cases), the lower limb salvage rate was 87.5% (28 in 32 cases). All patients who failed replantation underwent one or more surgeries after replantation, including amputation, debridement, flap transplantation, and vacuum sealing drainage (VSD).

**Table 1:** The severed part of 66 patients with replantation of severed limb

Severed part of limb	Number of patients
Forearm	21
Elbow	3
Upper arm	6
Shoulder	1
Forearm + upper arm	1
Wrist + forearm	2
Ankle	22
Shank	7
Thigh	3

**Table 2:** Clinical characteristics of 66 patients with replantation of severed limb

Characteristics	All patients (*n* = 66)	Failure (*n* = 48)	Success (*n* = 18)	*P*
Age, median (IQR), years	47.0 (36.0–54.5)	48.0 (38.0–56.0)	40.0 (28.5–52.5)	0.277
Sex, male patients, *n* (%)	48 (72.7)	38 (79.2)	10 (55.6)	0.108^b^
Traumatic condition				
Lower limb, *n* (%)	32 (48.5)	27 (56.3)	5 (27.8)	0.039
Blunt mutilation, *n* (%)	64 (97.0)	48 (72.7)	16 (24.2)	0.071^c^
Total mutilation, *n* (%)	29 (43.9)	22 (45.8)	7 (38.9)	0.613
Platelet count, × 10^9^/L, median (IQR)^a^	123.5 (68.5–195.3)	103.0 (60.0–178.0)	162.5 (101.3–245.3)	0.014
RBC count, × 10^12^/L, median (IQR)^a^	3.0 (2.5–3.8)	2.8 (2.3–3.4)	3.3 (3.0–4.1)	0.022
Albumin, g/L, median (IQR)^a^	27.0 (23.3–33.4)	25.8 (21.9–32.6)	32.7 (29.3–35.7)	0.002
Lactic acid on admission, mmol/L, median (IQR)	2.7 (1.2–4.6)	3.5 (1.4–4.8)	1.4 (0.9–3.0)	0.017
MESS, median (IQR)	10.0 (9.0–11.3)	11.0 (10.0–12.0)	9.0 (7.0–10.0)	< 0.001
Treatment and outcomes, median (IQR)				
Red cells suspension injected during surgery, mL	800.0 (375.0–1600.0)	850.0 (600.0–1750.0)	400.0 (0.0–900.0)	0.028
Hetastarch injected during surgery, mL	1000.0 (500.0–1500.0)	1000.0 (1000.0–1500.0)	1000.0 (500.0–1000.0)	0.015
72 h cumulative fluid balance after admission, mL	5101.5 (2761.2–7953.8)	5727.5 (3148.0–8589.0)	3041.0 (351.0–4663.2)	0.006
Length of hospital stay, day	51.5 (26.8–69.0)	57.0 (43.3–77.5)	25.0 (18.8–39.5)	< 0.001

*IQR* interquartile range, *RBC* red blood cell, *MESS* mangled extremity severity score

^a^First laboratory findings after surgery

^b^Yates’s correction was used

^c^Fisher’s exact test was used

Patients with lower limb amputation presented with a higher rate of replantation failure (56.3% of failure group vs. 27.8% of success group, *P* = 0.039) (Table 2). The failure group presented on admission higher median values of lactic acid (3.5 [IQR 1.4–4.8] vs. 1.4 [IQR 0.9–3.0] mmol/L, *P* = 0.017), MESS (11.0 [IQR 10.0–12.0] vs. 9.0 [IQR 7.0–10.0], *P* < 0.001), red cells suspension injected during surgery (850.0 [IQR 600.0–1750.0] vs. 400.0 [IQR 0.0–900.0] mL, *P* = 0.028), 72-h cumulative fluid balance after admission (5727.5 [IQR 3148.0–8589.0] vs. 3041.0 [IQR 351.0–4663.2] mL, *P* = 0.006), and length of hospital stay (57.0 [IQR 43.3–77.5] vs. 25.0 [IQR 18.8–39.5] days, *P* < 0.001). Patients in the failure group had more hetastarch injected during surgery (1000.0, IQR 1000.0–1500.0 mL) than those in the successful group (1000.0, IQR 500.0–1000.0 mL, *P* = 0.015) (Table 2).

Furthermore, the failure group had lower median values of red blood cell (RBC) count (2.8 [IQR 2.3–3.4] vs. 3.3 [IQR 3.0–4.1] × 10^12^/L, *P* = 0.022), platelet count (103.0 [IQR 60.0–178.0] vs. 162.5 [IQR 101.3–245.3] × 10^9^/L, *P* = 0.014), and albumin (25.8 [IQR 21.9–32.6] vs. 32.7 [IQR 29.3–35.7] g/L, *P* = 0.002) measured immediately after surgery (Table 2). No significant difference was observed in the length of ICU stay or other laboratory findings (Table S1 in Supplementary Material 1).

### Predictors and risk factors for replantation failure

**Fig. 2:**
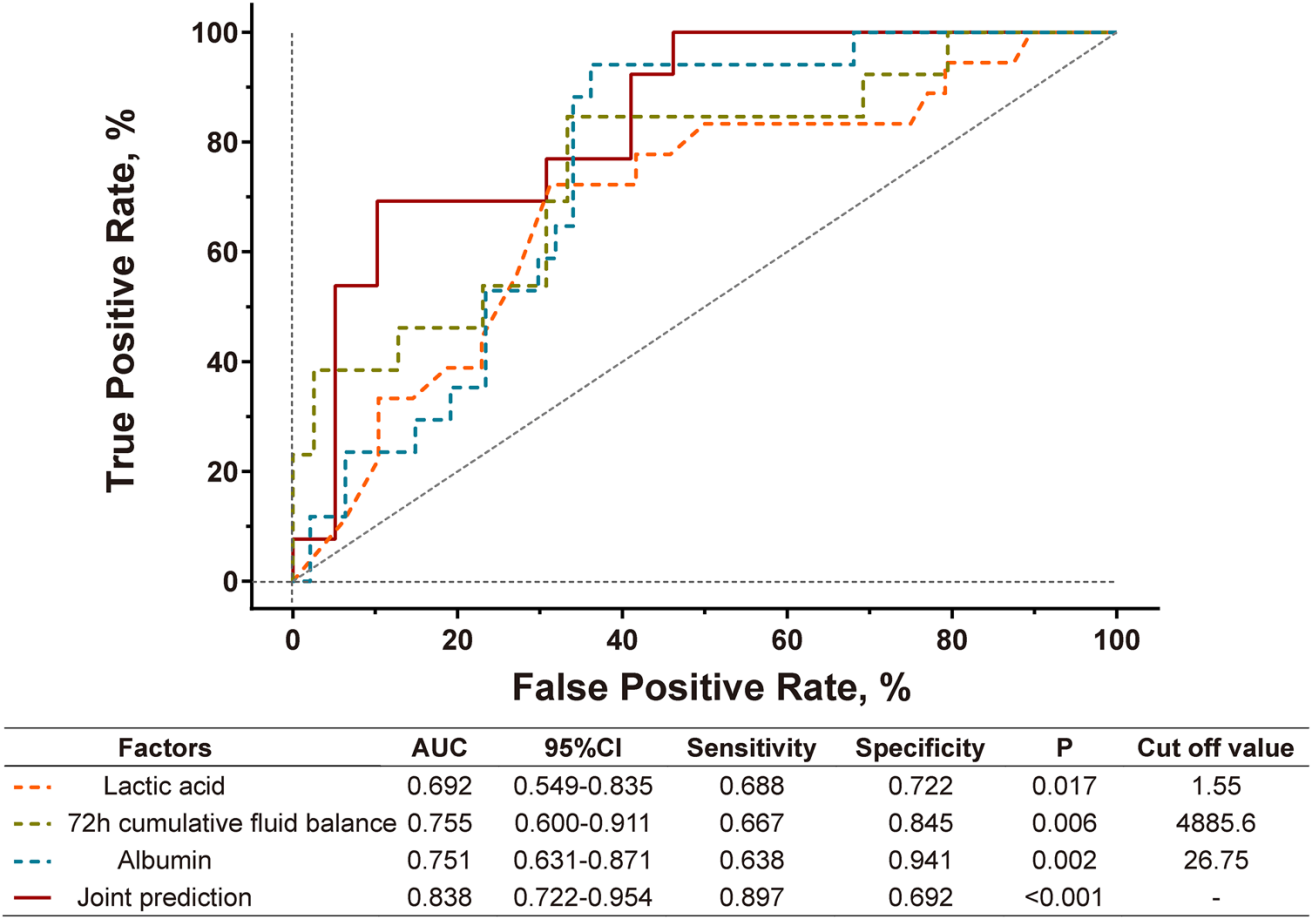
Combined indicators predict replantation failure. Lactic acid was measured on admission. Albumin level was measured after surgery immediately. Cutoff value was obtained through ROC analysis of each indicator

All patients underwent laboratory examinations after admission and surgery. The AUCs and cutoff values of each single index are shown in Table S2 (Supplementary Material 1). Combined predictors were selected according to the AUC, sensitivity, and specificity of each index. The results of the joint prediction analysis are presented in Table S3 (Supplementary Material 1). We found that three factors (lactic acid on admission, 72-h cumulative fluid balance, and albumin level immediately postoperatively) and their combined prediction showed predictive power for replantation failure (Fig. 2). The lactic acid cutoff on admission was 1.55 mmol/L with an AUC of 0.692 (95% confidence interval, 0.549–0.835; *P* = 0.017), a sensitivity of 68.8%, and a specificity of 72.2%. The 72-h cumulative fluid balance cutoff was 4885.6 mL with an AUC of 0.755 (95% CI 0.600–0.911; *P* = 0.006), a sensitivity of 66.7%, and a specificity of 84.5%. The immediate postoperative albumin level cutoff was 26.75 g/L with an AUC of 0.751 (95% CI 0.631–0.871; *P* = 0.002), a sensitivity of 63.8%, and a specificity of 94.1%. The AUC of joint prediction was 0.838 (95% CI 0.722–0.954; *P* < 0.001) with a sensitivity of 89.7% and a specificity of 69.2%.

**Fig. 3:**
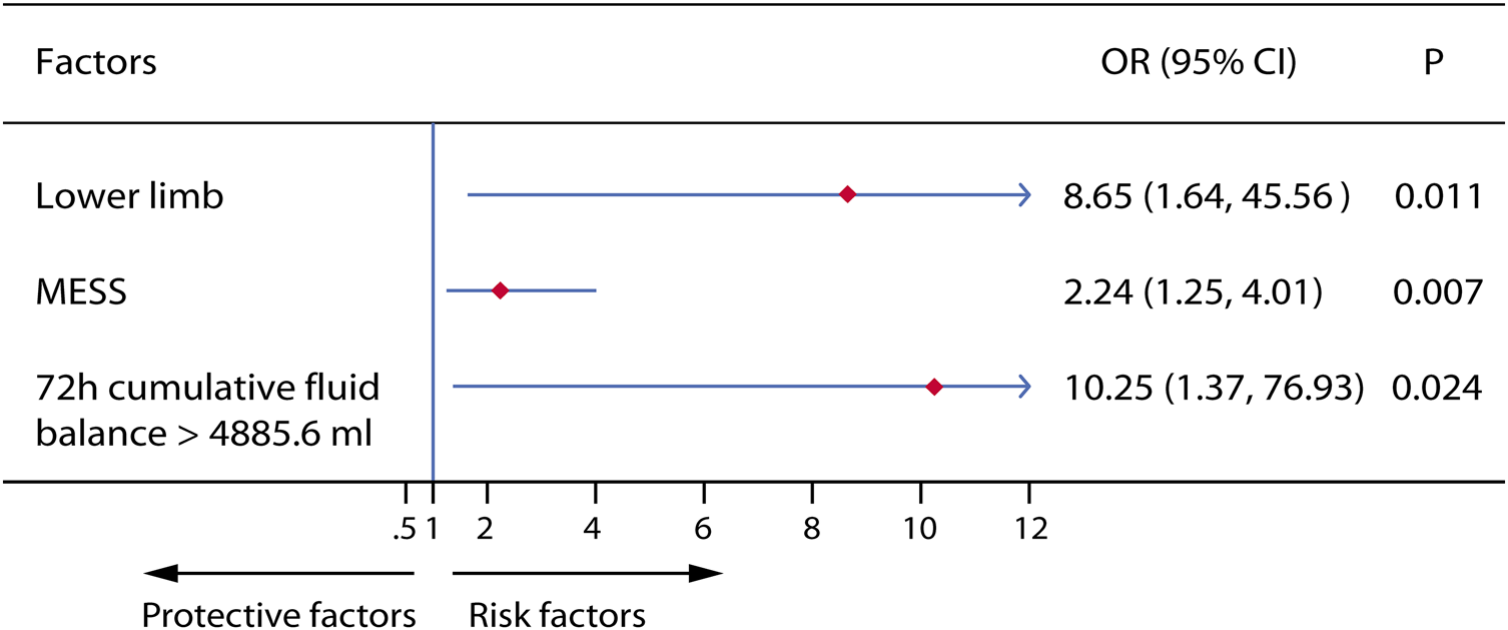
Multivariate logistic analysis of factors associated with replantation failure in 66 patients

Univariate logistic regression analysis showed that lower limb trauma, MESS, lactic acid on admission, RBC count immediately after surgery, platelet count after surgery, albumin level after surgery, volume of hetastarch injection during surgery, and 72-h cumulative fluid balance after admission were significantly associated with limb necrosis after replantation (Table S4 in Supplementary Material 1). Multivariable logistic regression analysis found that lower limb trauma (odds ratio [OR] 8.65, 95% confidence interval [CI] 1.64–45.56, *P* = 0.011), MESS (OR 2.24, 95% CI 1.25–4.01, *P* = 0.007), and first 72-h cumulative fluid balance > 4885.6 mL (OR 10.25, 95% CI 1.37–76.93, *P* = 0.024) were independent risk factors for replantation failure (Fig. 3).

## Discussion

In this retrospective study of patients with severe traumatic major limb mutilation who had replantation, we found that failure rate of replantation was 72.7%, and the delayed amputation rate was 10.6% (with a limb salvage rate of 89.4%). Lactic acid on admission, 72-h cumulative fluid balance, and albumin level taken immediately post-operation, and their joint prediction showed significant predictive power for replantation failure. Moreover, lower limb trauma, MESS, and first 72-h cumulative fluid balance > 4885.6 mL were independently associated with replantation failure.

The present results were similar to those of previous studies, which reported that the range of limb replantation (including fingers) failure rate was 45–86%, limb salvage rate was 40–85%, and the delayed amputation rate was 11.7–55.4%, among which the failure rate of complete mutilation of lower limbs and blunt injury were higher [22–26]. Higher injury severity, blunt trauma, location of trauma, and absence of pulses may be associated with failure [11]. All of the patients were in critical condition in our cohort, and almost all had blunt trauma accompanied by discontinuous vessels, nerves, muscles, and bone structures to varying degrees. High energy transfer from blunt injury has been noted to cause extensive damage to associated soft tissue, bone, and nerves [25, 27, 28], and this mechanism may be a potential cause of poor outcomes in this cohort.

### Lactic acid level

Lactic acid level was found to be one of the predictors of replantation failure in this study. Major limb mutilation is often accompanied by severe blood loss such that good perfusion is essential for graft survival [29]. Even mild preoperative anemia was associated with an increased risk of wounds, sepsis, and thromboembolic complications in patients undergoing major non-cardiac surgery [30]. Blood loss can result in oxygen delivery (DO_2_) reduction sufficient to cause tissue ischemia. Lactic acid may be helpful in predicting levels of oxygen debt accumulation and resuscitation needs, and its measurement may serve as a predictor of high-risk trauma [31]. Central venous oxygen saturation as another indicator of tissue oxygenation may be valuable for predicting the prognosis of large limb replantation [32].

### Hypoproteinemia

We also found that albumin levels were somewhat predictive of replantation failure. Albumin has the effect on maintaining fluid balance, and protect the microvasculature and mitigate increased vascular permeability via its antioxidant, anti-inflammatory effects, and anti-apoptotic effects [33, 34]. Hypoproteinemia is caused by bleeding and other factors [33], and is associated with inflammation and malnutrition [35]. Even there was no study on the association and mechanism between albumin level and replantation failure of major limbs, hypoproteinemia is associated with poor prognosis or complications of many diseases including the severity of the insult [33, 35, 36]. A low serum albumin concentration was reported as a prognostic factor after revascularization [37, 38]. However, it remains uncertain whether the effect of hypoalbuminemia on outcome is a cause–effect relationship or whether hypoalbuminemia is rather a marker of serious disease [33].These common laboratory indicators should be considered during diagnosis and treatment.

### Fluid balance-predictor and risk factor

Our results showed that the cumulative fluid balance had high sensitivity and good predictive ability. Fluid accumulation may lead to hemodilution, decreased perfusion pressure gradient due to elevated venous pressure, and inhibition of oxygen diffusion between capillaries and cells due to interstitial edema [39]. Microcirculatory hypoperfusion and organ ischemia–reperfusion injury are related to prolonged liquid administration time [40]. Reperfusion injury after replantation can lead to irreversible damage, which can activate complement, cytokines, and chemokines, resulting in cell, membrane, and microvascular damage that impairs outcomes [29]. This may partly explain the independent association between excessive fluid involvement and replantation failure.

### Lower limbs—the worse prognosis

Restoring tissue perfusion may be critical for successful limb salvage [19]. Of the tissues involved in major limb trauma, muscles are the least resistant to ischemia [41]. We found that lower limbs were independently associated with replantation failure, and had a lower salvage rate than upper limbs. Compared with the lower limbs, the upper extremities have less muscle mass, and an increase in collateral circulation may prolong the reperfusion time [42–44]. Anatomic and functional differences make the upper extremities more receptive to limb salvage and/or replantation than the lower limbs [45].

### MESS, still necessary?

The MESS evaluates limb trauma by integrating the extent of bone and soft tissue injury, limb ischemia, shock, and age [7]. Therapeutic advances in the treatment of vascular, orthopedic, neurologic, and soft tissue injuries have reduced the diagnostic accuracy of the MESS in predicting the need for amputation [7, 14]. That may explain MESS did not have a good sensitivity in predicting replantation failure in this study. Nevertheless, we found it was indeed an independent risk factor. The biological and clinical principles of MESS remain important, the score can reflect the extent of limb injury and are associated with poor prognosis [14, 46], and it may help quantify the overall severity of limb injury [11]. However, the optimal predictive value needs to be calibrated based on more researches, limb salvage and functional results [14].

Because of the limitations of its retrospective nature, this study could not obtain the details of trauma completely and could not evaluate the recovery of limb function of the patients. In the joint predictive analysis, the cutoff values of each single index could not be obtained. However, these indexes should still be considered in the traumatic mutilation of major limbs. Some indicators showed low sensitivity or specificity may because of the retrospective nature with small sample size. More prospective studies with a larger sample size are needed to further evaluate the robustness and values of the predictors, and to further analyze the potential predictors.

## Conclusion

Lower limb trauma, MESS, and 72-h cumulative fluid balance were associated with replantation failure. This implies the importance of fluid management in achieving major limb salvage. The combination of lactic acid, cumulative fluid balance, and albumin showed a significant predictive power for replantation failure. More studies are needed to explore the predictive power of indicators related to tissue oxygenation and wound healing for replantation failure.

## Supplementary Information

Below is the link to the electronic supplementary material.

Supplementary file1 (DOCX 31 KB) Table S1. Clinical characteristics of 66 patients with replantation of severed limb. Table S2. Predictors of failure after traumatic major limb replantation. Table S3. Combined indicators predict replantation failure. Table S4. Univariate logistic analysis of factors associated with replantation failure in 66 patients.

## Data Availability

Not applicable.
