# Supplementary material for: Risk and prognostic factors of replantation failure in patients with severe traumatic major limb mutilation
Source: Eur J Trauma Emerg Surg. 2022 Jan 20;48(4):3203–10. doi: 10.1007/s00068-021-01876-w (PMC9360147; doi:10.1007/s00068-021-01876-w)
Supplement: Supplementary file 1 — Supplementary file1 (DOCX 31 KB) Table S1. Clinical characteristics of 66 patients with replantation of severed limb. Table S2. Predictors of failure after traumatic major limb replantation. Table S3. Combined indicators predict replantation failure. Table S4. Univariate logistic analysis of factors associated with replantation failure in 66 patients. [file 68_2021_1876_MOESM1_ESM.docx]

Appendix

**Risk and prognostic factors of replantation failure in patients with severe traumatic major limb mutilation**

**Table S1.** **Clinical characteristics of 66 patients** **with replantation of severed limb.**

| **Characteristics** | **All patients**  **(n = 66)** | **Failure**  **(n = 48)** | **Success**  **(n = 18)** | **P** |
| --- | --- | --- | --- | --- |
| Pre-existing hypertension, n (%) | 7 (10.6) | 6 (12.5) | 1 (5.6) | 0.664 *^b^* |
| Pre-existing diabetes, n (%) | 4 (6.1) | 3 (6.3) | 1 (5.6) | 1.000 *^b^* |
| Current smokers, n (%) | 7 (10.6) | 4 (8.3) | 3 (16.7) | 0.380 *^b^* |
| Wound contamination |  |  |  | 0.189 |
| Mild, n (%) | 1 (1.5) | 0 (0.0) | 1 (5.6) |  |
| Moderate, n (%) | 29 (43.9) | 20 (41.7) | 9 (50.5) |  |
| Severe, n (%) | 36 (54.5) | 28 (58.3) | 8 (44.4) |  |
| Time from trauma to admission, hr., median (IQR) | 3.0 (1.0-4.0) | 3.0 (1.0-4.1) | 2.8 (1.2-4.0) | 0.942 |
| Time from trauma to operation started, hr., median (IQR) | 3.7 (3.0-5.5) | 3.7 (3.0-5.5) | 3.9 (2.7-5.6) | 0.768 |
| First laboratory findings after surgery, median (IQR) |  |  |  |  |
| WBC count, × 109/L | 10.2 (8.3-13.5) | 10.2 (8.3-13.5) | 10.4 (6.7-13.6) | 0.666 |
| Neutrophil count, × 109/L | 9.0 (6.8-12.2) | 9.1 (7.1-12.3) | 8.9 (5.1-11.8) | 0.433 |
| Lymphocyte count, × 109/L | 0.6 (0.5-1.1) | 0.6 (0.4-1.0) | 0.6 (0.6-2.2) | 0.092 |
| Prealbumin, g/L | 188.7  (144.0-240.7) | 177.5  (134.5-224.6) | 203.7  (166.0-252.9) | 0.056 |
| BUN, mmol/L | 4.6 (3.8-5.9) | 4.9 (3.8-6.0) | 4.3 (3.3-5.8) | 0.327 |
| Creatinine, μmol/L | 58.3 (50.7-70.7) | 58.5 (51.8-72.7) | 58.0 (46.1-64.1) | 0.224 |
| Treatment and outcomes, median (IQR) |  |  |  |  |
| Duration of operation, hr. | 6.5 (4.5-8.0) | 6.9 (4.8-8.2) | 6.3 (3.9-7.5) | 0.187 |
| Ischemia time, hr. *^a^* | 7.5 (6.2-9.5) | 7.5 (6.6-9.5) | 6.8 (5.5-8.5) | 0.132 |
| FFP injected during surgery, ml | 400.0  (0.0-643.8) | 400.0  (200.0-793.8) | 250.0  (0.0-425.0) | 0.072 |
| Crystal solution injected during surgery, ml | 1900.0  (1500.0-2737.5) | 2000.0  (1500.0-3000.0) | 1500.0  (1237.5-2125.0) | 0.053 |
| Length of ICU stay, day | 8.0 (5.0-11.0) | 9.0 (5.0-13.0) | 5.0 (4.0-9.0) | 0.059 |

*^a^* Ischemia time was defined as the time from mutilation to recovery of blood circulation.

*^b^* Fisher’s exact test was used.

Abbreviations: IQR, interquartile range; WBC, white blood cell; BUN, blood urea nitrogen; FFP, fresh frozen plasma; ICU, intensive care unit.

**Table S2. Predictors of failure after traumatic major limb replantation.**

| **Factor** | **AUC (95% CI)** | **Sensitivity** | **Specificity** | **Cut-off value** | **P** |
| --- | --- | --- | --- | --- | --- |
| MESS | 0.831 (0.734-0.928) | 0.542 | 1 | 10.5 | <0.001 |
| RBC count, 10 × 10^9^/L *^a^* | 0.685 (0.556-0.813) | 0.458 | 0.889 | 2.75 | 0.022 |
| Platelet count, × 10^9^/L *^a^* | 0.698 (0.559-0.836) | 0.625 | 0.722 | 141.0 | 0.014 |
| Albumin, g/L *^a^* | 0.751 (0.631-0.871) | 0.638 | 0.941 | 26.75 | 0.002 |
| Lactic acid on admission, mmol/L | 0.692 (0.549-0.835) | 0.688 | 0.722 | 1.55 | 0.017 |
| 72 hours cumulative fluid balance after admission, ml | 0.755 (0.600-0.911) | 0.667 | 0.845 | 4885.6 | 0.006 |

*^a^* First laboratory findings after surgery.

Abbreviations: MESS, mangled extremity severity score; RBC, red blood cell.

**Table S3.** **Combined indicators predict replantation failure.**

| **Factors** | **AUC (95% CI)** | **Sensitivity** | **Specificity** | **P** |
| --- | --- | --- | --- | --- |
| 72 hours cumulative fluid balance, lactic acid, albumin, platelet | 0.836 (0.719-0.953) | 0.897 | 0.692 | <0.001 |
| 72 hours cumulative fluid balance, lactic acid, albumin | 0.838 (0.722-0.954) | 0.897 | 0.692 | <0.001 |
| 72 hours cumulative fluid balance, albumin, platelet | 0.832 (0.712-0.952) | 0.769 | 0.692 | <0.001 |
| 72 hours cumulative fluid balance, lactic acid, platelet | 0.769 (0.596-0.943) | 0.872 | 0.692 | 0.004 |
| Lactic acid, albumin, platelet | 0.776 (0.659-0.892) | 0.660 | 0.824 | 0.001 |
| 72 hours cumulative fluid balance, albumin | 0.826 (0.704-0.949) | 0.769 | 0.769 | <0.001 |
| 72 hours cumulative fluid balance, lactic acid | 0.777 (0.614-0.940) | 0.897 | 0.692 | 0.003 |
| 72 hours cumulative fluid balance, platelet | 0.748 (0.588-0.907) | 0.949 | 0.462 | 0.008 |
| Lactic acid, albumin | 0.757 (0.638-0.877) | 0.574 | 0.941 | 0.002 |
| Albumin, platelet | 0.782 (0.671-0.894) | 0.617 | 0.992 | 0.001 |
| Lactic acid, platelet | 0.712 (0.563-0.860) | 0.708 | 0.778 | 0.008 |

**Table S4. Univariate logistic analysis of factors associated with replantation failure in 66 patients.**

| **Factor** | **Unadjusted OR (95% CI)** | **P** |
| --- | --- | --- |
| Age, years | 1.02 (0.98-1.06) | 0.278 |
| Male, sex | 3.04 (0.95-9.71) | 0.061 |
| Time from trauma to admission, hr. | 0.98 (0.69-1.37) | 0.885 |
| Time from trauma to operation started, hr. | 1.05 (0.77-1.43) | 0.773 |
| Lower limb | 3.34 (1. 03-10.86) | 0.045 |
| MESS | 2.17 (1.41-3.33) | <0.001 |
| RBC count, × 10^9^/L *^a^* | 0.57 (0.32-0.99) | 0.045 |
| Platelet count, × 10^9^/L *^a^* | 0.99 (0.98-0.99) | 0.012 |
| Albumin, g/L *^a^* | 0.86 (0.77-0.96) | 0.005 |
| Fibrinogen, mg/dl *^a^* | 0.43 (0.15-1.27) | 0.127 |
| D-dimer, ng/ml | 0.94 (0.73-1.21) | 0.636 |
| Lactic acid on admission, mmol/L | 1.43 (1.03-1.98) | 0.032 |
| Ischemia time, hr. | 1.19 (0.92-1.54) | 0.195 |
| Hetastarch injected during surgery, ml | 6.77 (1.40-32.73) | 0.017 |
| 72 hours cumulative fluid balance after admission > 4885.6 ml | 9.46 (1.96-45.71) | 0.005 |

*^a^* First laboratory findings after surgery.

Abbreviations: MESS, mangled extremity severity score; RBC, red blood cell; OR, odds ratio.
